# Effect of Chinese medicine prescription on nephrotic syndrome: A protocol for systematic review and meta-analysis

**DOI:** 10.1097/MD.0000000000020622

**Published:** 2020-06-05

**Authors:** Yanli Deng, Leixiao Zhang, Aiwei Wen, Dongxian Xu, Wenping Wang, Yuhao Hou, Zhen Liu, Lin Yang, Tao Shen, Qin Luo, Wei Wu, Yuanshu Ou

**Affiliations:** a Nephropathy Department, Sichuan Second Chinese Medicine Hospital; b Acupuncture and Tuina School, Chengdu University of Traditional Chinese Medicine, Chengdu, Sichuan, China.

**Keywords:** Chinese medicine prescription, Nephrotic syndrome, protocol, systematic review

## Abstract

**Background:**

Nephrotic syndrome (NS) is a common chronic recurrent kidney disease. Many trials have shown that Chinese medicine prescription (CMP) can effectively treat NS. The program aims to evaluate the efficacy and safety of CMP for NS.

**Methods:**

This systematic evaluation will entail an electronic and manual search of all CMP for NS from inception to February, 2020, regardless of the publication status or language. Databases include PubMed, Embase, Springer, Web of Science, the Cochrane Library, the World Health Organization International Clinical Trial Registration Platform, the Chinese Medicine Database, the China National Knowledge Infrastructure, the Chinese Biomedical Literature Database, the China Science Journal Database, and the Wanfang Database. Other sources of information, including bibliographies and meeting minutes for identified publications, will also be searched. A manual search for grey literature, including unpublished conference articles will be performed. Additionally, any clinical randomized controlled trials related to CMP for NS, regardless of the publication status and language limitations, will be included in the study. Study selection, data extraction, and research quality assessments will be conducted independently by 2 researchers. The main result was the total clinical efficacy rate or other validated scales after at least 2 weeks of treatment. Secondary outcomes included 24-hour urine protein quantification, blood urea nitrogen, serum creatinine, C-reactive protein, tumor necrosis factor-α, and interleukin 6, recurrence rates and adverse events during follow-up. Implement the Cochrane RevMan V5.3 bias assessment tool to assess bias risk, data integration risk, meta-analysis risk, and subgroup analysis risk (if conditions are met). Mean difference, standard mean deviation and binary data will be used to represent continuous results.

**Results:**

This study will provide a comprehensive review and evaluation of CMP for the treatment of NS.

**Conclusion:**

This study will provide new evidence for evaluating the effectiveness and side effects of CMP on NS. Since the data is not personalized, formal ethical approval is not required.

**INPLASY registration number:**

INPLASY202040181.

## 1 Introduction

### 1.1 Description of the condition

Nephrotic syndrome (NS) is a rare diseases (around 2–7 cases per 100,000 children per year) characterized by proteinuria ≥50 mg/kg/day (or ≥40 mg/m^2^/h) or a proteinuria/creatininuria ratio >2 (mg/mg); hypoalbuminemia less than 25 g/L and edema.^[1,2]^ As a result of massive proteinuria and hypoalbuminemia, this syndrome is frequently accompanied by edema, dyslipidemia, abnormalities in coagulation/fibrinolysis, reduced renal function, and immunological disorders. It is caused by increased permeability of serum protein through the damaged basement membrane in the renal glomerulus.^[3,4]^ A wide range of primary or secondary renal diseases can cause NS. One of the main complications of NS is venous thrombosis in the deep veins of the lower limbs.^[5]^ Other complications are end stage kidney disease, infections, and cardiovascular events.^[6]^ The protein leakage, with the consequent hypoalbuminemia and edema, due to podocyte alterations may be caused by genetic diseases, immunological mechanisms, infections, toxins or malignancy.^[1]^

Histologically, NS can be caused due to membranous glomerulonephritis, focal segmental glomerulosclerosis, diffuse mesangial sclerosis, or minimal change disease (MCD).^[7]^ A common cause of NS in adults is membranous nephropathy and in children minimal change nephropathy.^[8]^ The total cases of membranous glomerulonephritis and MCD were close to 80% among the primary glomerular diseases. In the analysis of NS patients aged C65 years, the ratios of diabetic nephropathy and amyloid nephropathy were highest, next to primary glomerular disease. MCD shows a higher remission rate of C90%, whereas the recurrence rate is also higher at 30% to 70%. In the data in Japan, the renal survival rate was 33.5% at the 20-year follow-up examination.^[3]^ The current use for more than 2 weeks, recent use, and past use (>2 months to 2 years) of conventional Non-Steroidal Antiinflammatory Drugs were associated with a higher risk of NS.^[9]^ The epidemiological status of NS varies in different countries and regions. The average global incidence of NS is 40.35%, with the highest rate in Iran (70%) and the lowest in Finland (16.4%). With the aging of the Chinese population, the incidence of NS is also increasing, accounting for 42.2%. Cases of NS within the same family are relatively rare; most cases are isolated, with no family history.^[10]^

Patients with NS show various urinary abnormalities and renal dysfunction. The degrees of proteinuria and hematuria differ with each histological type of NS. High urinary specific gravity and various kinds of cast formation, including hyaline, granular, waxy, and fatty, are frequently noticed in NS.^[3]^ In the early phase, edema appears in local parts such as the eyelids; in the advanced phase, generalized edema occurs with pleural effusion and ascites.

Due to its complicated pathogeny and various pathological types, treatment of NS is very difficult in the clinic.^[11]^ Salt restriction is essential for the alleviation of edema in NS.^[3]^ The published guideline from the Japanese Society of Nephrology, Guidelines of lifestyle and diet therapy for patients with chronic kidney disease, recommends a protein intake of 1.0 to 1.1 g/kg body weight (BW)/day in minimal change NS and 0.8 g/kg BW/day in other NSs. To keep the nitrogen balance, a calorie intake of 35 kcal/kg BW/day is recommended. At present, the treatment of NS is mainly based on oral Angiotensin-Converting Enzyme Inhibitors, glucocorticoids, immunosuppressants and other drugs.^[8,12]^ Oral steroid therapy is usually administered as the initial treatment for minimal change NS. In the evaluation of efficacy, a high response rate of 90% was found. Steroid pulse therapy may be considered when absorption of oral steroids seems difficult because of intestinal edema, diarrhea, and other conditions.^[3]^ The response to the treatment is variable among different ethnics and populations and resistance to the steroids is detected in almost 50% of adult patients.^[7]^ However, the shortcomings of long treatment duration, easy relapse, hormone dependence and obvious toxic and side effects cannot be ignored.^[12]^ Immunosuppressant therapy can induce many serious side effects, such as diverse organ failure (cardiac, renal, and ear), toxicities,^[13]^ fungal infection and a fast relapse following any stoppage in therapy.^[14]^

NS has the characteristics of easy recurrence and low complete remission rate. Without treatment, one third of patients with membranous nephropathy achieve spontaneous remission, one third develop persistent proteinuria and one third progress to end stage kidney disease.^[15]^ Clearly, the present pharmaceutical treatments are not sufficient for patient to prevent NS from developing into renal failure. At end-stage, dialysis and kidney transplantation are the final options for the patients.^[16]^

The curative and protective effects of traditional Chinese medicine (TCM) have already been widely and fully recognized through clinical practice for thousands of years in China.^[17]^ Compared with modern drugs, CMP had some special advantages in treating NS, especially in the aspects of reducing proteinuria and side effects of immunosuppressive drugs, as well as alleviating the complications of hypercoagulability or gastrointestinal mucosa edema.^[18,19]^ Furthermore, compared with hormone, CMP application to treatment of NS is also a good way to avoid side effects and to reduce high recurrence rate of disease.^[17]^ Therefore, in this paper, we will systematically review and critically evaluate published randomized controlled trials (RCTs) by comparing CMP and various types of control interventions to determine the effectiveness and safety of CMP.

### 1.2 Description of the intervention

The most characteristic clinical manifestation of NS is edema. According to the theory of Chinese medicine, the reason for edema could be concluded to Qi Xu, especially caused by deficient spleen or kidney functions. In other words, the disorder of the spleen or kidney is an important factor during NS development. A large number of literatures indicated that CMP showed a significant effect on NS.^[20,21]^

Fangji Huangqi Tang, a classic TCM prescription in Chinese clinical application for the treatment of NS, is composed of the roots of 4 kinds of Chinese herbal medicines, namely, *Stephania tetrandra* S. Moore (FJ), *Astragalus membranaceus* Fisch. ex Bunge (HQ), *Atractylodes macrocephala* Koidz. (BZ), and *Glycyrrhiza uralensis* Fisch. ex DC. (GC).^[17]^ Fangchinoline and tetrandrine were proved to be the main effective chemical materials of Fangji Huangqi Tang to cure NS in rats.^[20]^ Qi-Dan Fang consists of two of the most extensively applied herbal remedies among Traditional Chinese Medicine (TCM) (*Radix Astragali Mongolici* and *Radix Salviae Miltiorrhizae*, with a weight ratio of 5:1) which are specifically used for the treatment of various kidney diseases.^[18]^*Radix Astragali* does not only decrease serum lipids including total cholesterol, triglycerides, low-density lipoprotein cholesterol, but also does improve serum albumin levels, plasma albumin levels, and ameliorate blood protein metabolism disorder symptoms of NS.^[22]^*Radix Salviae Miltiorrhizae* could suppress pain, activate blood circulation, eliminate stasis, and improve blood function, which has been proven to protect the kidneys in many experiments and clinical researches.^[23,24]^ There is no doubt that Chinese herbal compounds, a combined drug treatment of 2 or more compounds, could offer beneficial synergistic relief effects for NS.^[18]^

### 1.3 Objectives

To develop treatment recommendations, we systematically evaluated the efficacy and safety of CMP for NS.

## 2 Methods

### 2.1 Study registration

INPLASY registration number is INPLASY202040181. This protocol report is structured according to the Preferred Reporting Items for Systematic Reviews and Meta-Analyses Protocols (PRISMA-P) statement guidelines.^[25]^ The review will be conducted in accordance with the PRISMA guidelines and follows the recommendations of the Cochrane Handbook for Systematic Reviews of Interventions.^[26,27]^ If we refine the procedures described in this protocol, we will update the record in the INPLASY and disclose them in future publications related to this study (https://inplasy.com/).

### 2.2 Inclusion criteria for study selection

#### 2.2.1 Types of study

To evaluate the curative effects of CMP on NS, this review is confined to RCTs comparing of CMP with a control group, which conventional western medicine, including hormones, immunosuppressive agents, diuretics, antiplatelet aggregation drugs, Chinese patent medicines, and dietary maintenance. It is deemed a randomized study if the trial stated the randomization phrase, and the blinding is not restricted. The language will be limited to Chinese and English. The animal mechanism studies, case reports, self-pre- and post-control, or non-RCTs are excluded.

#### 2.2.2 Types of participants

Regardless of gender, age, ethnicity, education, and economic status, patients with nephrotic syndrome who meet the following diagnostic criteria (eg, Evidence-based clinical practice guidelines for nephrotic syndrome 2014) will be included.^[3]^

#### 2.2.3 Types of intervention

The review comprises clinical trials with the treatment of CMP. The intervention measures of the experimental group were CMP alone or CMP combined with conventional western medicine, regardless of the dosage form (tablets, mixtures, decoctions). We will evaluate and compare CMP based on the training and education of physicians, their clinical experience, the total number of CMP treatments, the duration and frequency of treatment, etc.

#### 2.2.4 Types of outcome measures

The primary outcome was the effect of CMP on NS using total clinical efficacy rate or other validated scales, after at least two weeks of treatment. Secondary outcomes include 24-hour urine protein quantification, blood urea nitrogen, serum creatinine, C-reactive protein, tumor necrosis factor-α, and interleukin 6. Recurrence rates and adverse events during follow-up.

### 2.3 Data sources

We will search electronically and manually for all RCTs that treat traditional Chinese medicine prescriptions for nephrotic syndrome, regardless of publication status and language, up to February 2020. Databases include: PubMed, Embase, Springer, Web of Science, Cochrane Library, WHO International Clinical Trials Registry Platform (ICTRP), Traditional Chinese Medicine databases (TCMD), China National Knowledge Infrastructure (CNKI), China Biomedical Literature Database (CBM), Chinese Scientific Journal Database (VIP), and Wan-Fang database. Other sources, including reference lists of identified publications and meeting minutes, will also be searched. Manually search for grey literature, including unpublished conference articles.

### 2.4 Search strategy

The search strategy will be followed the PRISMA guidelines. The key search terms are ("nephrotic syndrome” or "nephrotic”) and ("Chinese medicine prescription” or "prescription”) and (“randomized”). The search strategy will be adapted to different databases demands. Search strategy in PubMed is shown in Table 1.

**Table 1:**
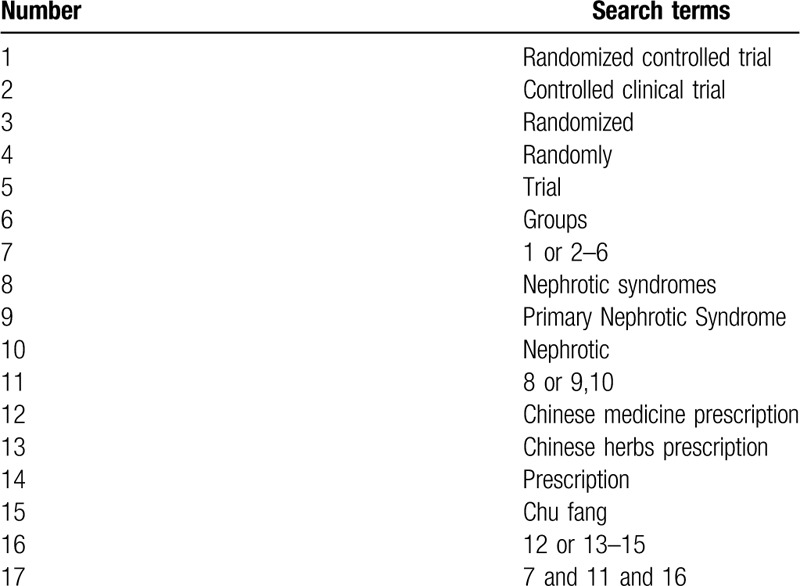
PubMed search strategy.

### 2.5 Data collection and analysis

#### 2.5.1 Selection of studies

**Figure 1:**
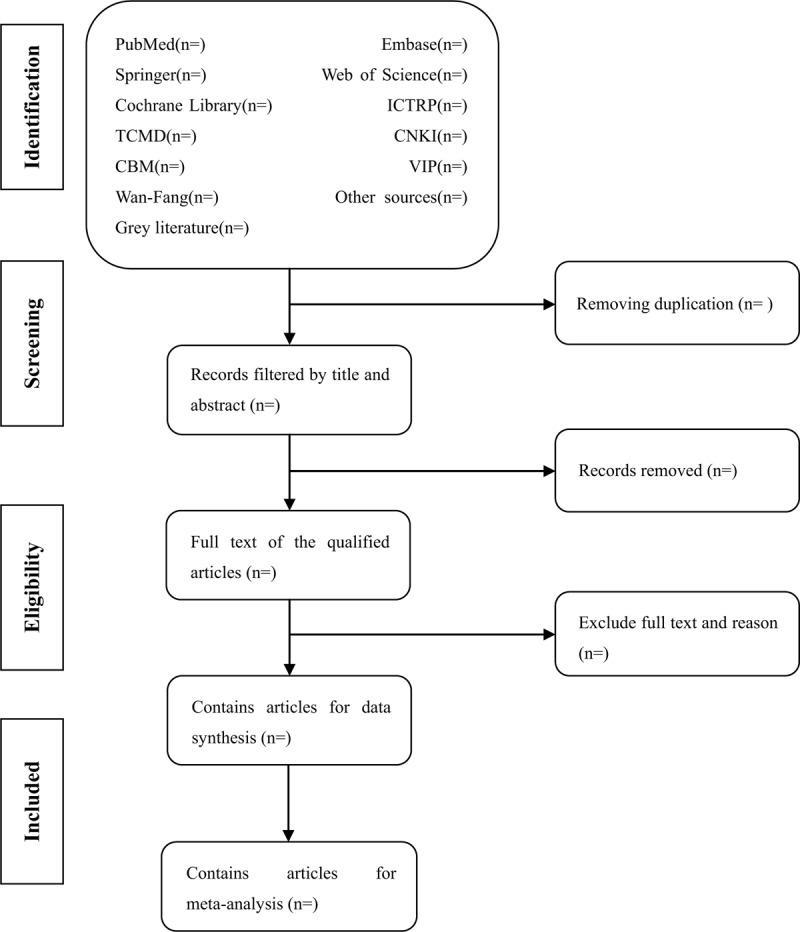
Flow diagram of studies identified.

Prior to literature retrieval, all reviewers are trained to ensure a basic understanding of the background and purpose of the review. In the literature screening process, we will use EndNote software (V.X8) document management software. The 2 comment authors (YLD and LXZ) will be in strict accordance with the inclusion criteria, independent screen all retrieval research, read the title, abstract and key words in the literature, and determine which meet the inclusion criteria. We will obtain the full text of all relevant studies for further evaluation. Excluded studies will be documented and explained. If there is a disagreement in the selection process, it will be discussed by the 2 authors (YLD and LXZ) and the third author (YSO) and arbitrated if necessary. If necessary, we will contact the trial author for clarification. The primary selection process is shown in a PRISMA flow chart (Fig. 1).

#### 2.5.2 Data extraction and management

The authors will extract data independently from the selected report or study and fill out the data extraction form. We will obtain data on general information, participants, methods, interventions, outcomes, results, adverse events, conflicts of interest, ethical recognition, and other information. For publications with insufficient or ambiguous data, we will attempt to obtain information from the corresponding authors by e-mail or telephone. Any differences will be resolved through discussions between the 2 authors, and further differences will be arbitrated by the third author (YSO).

#### 2.5.3 Assessment of risk of bias and reporting of study quality

The authors (AWW and DXX) will use the Cochrane Collaboration's bias risk assessment tool to assess the risk of bias in all included studies. We will assess the risk of bias in the following areas: sequence generation, assignment sequence hiding, the blindness of participants and staff, and result evaluators, incomplete outcome data, selective outcome reporting, and other sources of bias. This review uses L, U, and H as the key to these assessments, where L (low) indicates a lower risk of bias, U (unclear) indicates that the risk of bias is uncertain, and H (high) indicates a higher risk of bias. If inconsistent results appear, the final decisions will be made by the third author (YSO). Information on the risk of biased assessments included in the study is summarized in tabular form and the results and impacts are critically discussed. If the information is ambiguous, we will try to contact the author. For repeated publications, we only select the original text.

#### 2.5.4 Measures of treatment effect

Data analysis and quantitative data synthesis will be performed using RevMan V.5.3. For continuous data, if there is no heterogeneity, we will use mean difference or standard mean difference to measure the therapeutic effect of 95% confidence interval (CI). If significant heterogeneity is found, a random effects model will be used. For dichotomous data, we will use the 95% CI risk ratio for analysis.

#### 2.5.5 Unit of analysis issues

We will include data from parallel group design studies for meta-analysis. Only the first phase of the data will be included in the random crossover trial. In these trials, participants were randomly divided into two intervention groups and individual measurements for each outcome of each participant were collected and analyzed.

#### 2.5.6 Management of missing data

If the primary result has missing or incomplete data, we will contact the author of the communication to obtain the missing data. If it is never available, exclude the experiment from the analysis.

#### 2.5.7 Assessment of heterogeneity

We will use the Review Manager to assess efficacy and publication bias (version 5.3, Nordic Cochrane Centre, Copenhagen, Denmark). The forest map is used to illustrate the relative strength of the effect. The funnel plot is used to illustrate the bias because the number of trials exceeds 10. If a significant difference is detected, a random effects model will be used.

#### 2.5.8 Assessment of reporting biases

We will use a funnel plot to detect report bias. If more than 10 trials are included, the funnel plot will be used to assess the reported bias. If the funnel plot is found to be asymmetrical, analyze the cause using Egger's method. We will include all eligible trials regardless of the quality of the method.

#### 2.5.9 Data synthesis

We will use RevMan for all statistical analysis. If considerable heterogeneity is observed, a 95% CI random effects model will be used to analyze the combined effect estimates. Subgroup analysis will be performed with careful consideration of each subgroup if necessary.

#### 2.5.10 Subgroup analysis

There is no presubgroup plan. Subgroup analysis was performed based on control interventions and different outcomes.

#### 2.5.11 Sensitivity analysis

Based on sample size, heterogeneity quality, and statistical models (random or fixed-effect models), we will perform sensitivity analysis.

#### 2.5.12 Grading the quality of evidence

The quality of evidence for all outcomes will be judged by the Grading of Recommendations Assessment, Development, and Evaluation (GRADE) working group approach. Bias risk, consistency, directness, precision, publication bias, are aspects of our assessment. High, medium, low or very low represents the 4 levels of evaluation.

## 3 Discussion

Globally, more and more patients are affected by NS, which places a huge burden on global healthcare resources. Early detection and prevention and effective treatment can reduce the global burden and improve the quality of life of patients. The evaluation of this systematic review will be divided into 4 parts: identification, the inclusion of literature, data extraction, and comprehensive analysis of data. According to the Cochrane method, this study is based on the analysis of clinical RCT evidence at home and abroad, searching and screening the main electronic literature database with evidence-based medical evidence, providing clinicians with more convincing evidence in decision-making, to better guide clinical treatment.

## Author contributions

**Conceptualization:** Y. Deng, W. Wu, Y. Ou

**Methodology:** L. Zhang, Q. Luo

**Software:** A. Wen, D. Xu, Y. Hou

**Supervision:** W. Wang, Z. Liu

**Validation:** L. Yang, T. Shen

**Writing – original draft:** Y. Deng, L. Zhang
